# Prophylaxis

**Published:** 1911-03-15

**Authors:** Edward C. Kirk


### PROPHYLAXIS.

BY EDWARD C. KIRK, D.D.S. SC.D.

Prophylaxis is broadly defined as "the prevention of disease—preventive medicine." In its relation to dentistry the application of the prophylaxis idea has thus far been dominated by adherence to a certain principle quite analogous to that which limited its application in medicine some years ago, but which is today largely superseded by a broader and more comprehensive conception. Following the demonstration of the truth of the germ theory of disease causation by the work of Koch and his co-laborers thirty years ago, the prophylactic idea of disease prevention was focused upon ways and means for antiseptically exterminating germs that were the known exciters of disease action. Hence followed the long period of research into the nature and mode of action of germicidal and antiseptic agents, a research which not only brought forth a marvelous accumulation of data as to known antiseptics and germicidal agents, but stimulated the production of new ones and developed a corresponding fund of information concerning the nature of bacteria and the diseases caused by them.

Incidently, much was brought to light as to the limitations of bactericidal therapy through the medium of chemical substances, for it was quickly found that practically all agents having a potentiality great enough to destroy the vitality of the micro-organisms of disease were sufficiently powerful to act similarly upon the cellular elements of the tissues—a conclusion that greatly limited the use of antiseptic substances in diseases due to deep invasions of pathogenic organisms, especially those of high virulency.

The inadequacy of germicidal substances for the treatment of the more virulent infections stimulated investigation of the defensive forces of the normal organisms, and gradually brought to light the factors that now constitute our knowledge of susceptibility and immunity, together with the enormously important fact that these two states, variable with the individual and as among individuals, are not only directly related to the nutritional processes, but may be within certain limitations increased or diminished by artificial means;—out of which discovery has grown the most recent triumph of medical research, viz, serum therapy, which not only bids fair to revolutionize the whole science and art of therapeutics, but the principles upon which it is based seem destined to become the sheet anchor of scientific prophylaxis.

As investigation proceeds, the general fact is becoming more and more evident that susceptibility to bacterial invasion and consequent disease reaction upon the part of the organism is the resultant of defective resistance upon the part of the invaded tissues, and that whatever may be found to be the ultimate chemical and physical nature of the defensive substances in the tissues of the immune individual, they are nutritional products—something elaborated by the organism itself; and that when through any cause the production of these defensive substances falls below normal invasion of pathogenic bacteria is prone to occur. The maintenance of health and resistance to disease is therefore a nutritional problem, and so far as pathogenic bacteria are concerned, prophylaxis becomes a question of defense rather than of offence against them.

When the application of these main deductions of scientific research in bacterio-pathology and therapeutics is made to the conditions met with in the human mouth, there seems to be room for a much broader definition of dental and oral prophylaxis than has yet been practically made in that connection. The catchy formula that "Clean teeth will not decay" is misleading, even if true in the ideal sense. Not only is it misleading, but like many other adage it is obstructtive of further progress—for two reasons: first, because it is equally true that unclean teeth do not necssarily decay; and second, it is practically impossible to always have clean teeth in the ideal sense. The phrase is obstructive of further progress because it concentrates our attention upon an erroneous conception as to the causes of tooth decay. For the reasons just stated dental carries cannot logically or scientifically, any more than from the standpoint of observed facts, be classified as a filth disease. There are the best reasons why the mouth should be kept as clean as modern prophylactic methods are capable of accomplishing that desirable and necessary result, but we must search farther back than a dirty mouth for the cause of dental caries. To that end we have on various occasions directed attention in these pages the close relationship between the composition of the bodily juices, the salivary composition, etc., and the food habit of the individual, and dental caries. More particularly has attention been called to the fact that the fermenting particles of carbohydrate food debris adhering to tooth surfaces is not of itself sufficient to account for the phenomena of caries of the teeth; that the mixed saliva itself contains fermentable substances as it is excreted from the glands, and that this carbohydrate product of nutrition dialyzed from the blood plasma is the element found in the saliva of caries susceptibles which is the normal pabulum of the lactic acid bacteria concerned in the first stages of tooth caries. It is in the direction of salivary composition that the true solution of the etiology of dental decay is to be sought out and, we believe, found. But as salivary composition is a resultant of nutrition, and that in its turn is dependent in large degree upon food habit, the true prophylaxis of dental caries will come through correction and balancing of the food ration rather than through the altogether artificial and empirical use of the tooth-brush—which, in the light of rationalism, should take its place with other implements and devices of the toilet for the ends of bodily cleanliness, and be no longer regarded as the sum total of our weapons of defense against dental caries.

The present world-wide activity of interest in dental and oral hygiene should include as among the most important factors of its research work among school children an investigation of the question of their food habits, and subsequently correlate the data with respect to that factor with observations upon the prevalence of dental caries. Statistics covering the relation of food habit have not been extensively collected in the light of modern studies of the general nutritional question, and data of that character are more needed now than additions to our knowledge of the prevalence of caries. We know that caries is universal what we now need to know is why it is universal, so that we may happily, from the solution of that fundamental problem, devise something worthy to be called prophylaxis in the larger meaning of that term.—*Dental Cosmos.—February* 1911.

